# *Helicobacter pylori cagA* status and gastric mucosa-associated lymphoid tissue lymphoma: a systematic review and meta-analysis

**DOI:** 10.1186/s41043-021-00280-9

**Published:** 2022-01-03

**Authors:** Masoud Keikha, Amirhossein Sahebkar, Yoshio Yamaoka, Mohsen Karbalaei

**Affiliations:** 1 Department of Microbiology and Virology, School of Medicine, Mashhad University of Medical Sciences, Mashhad, Iran; 2 Biotechnology Research Center, Pharmaceutical Technology Institute, Mashhad University of Medical Sciences, Mashhad, Iran; 3 Applied Biomedical Research Center, Mashhad University of Medical Sciences, Mashhad, Iran; 4 Department of Environmental and Preventive Medicine, Faculty of Medicine, Oita University, Yufu, Oita Japan; 5 Global Oita Medical Advanced Research Center for Health, Oita University, Yufu, Oita Japan; 6 Department of Microbiology and Virology, School of Medicine, Jiroft University of Medical Sciences, Jiroft, Iran

**Keywords:** CagA antigen, Gastric MALT lymphoma, *Helicobacter pylori*, Meta-analysis

## Abstract

**Background:**

Recent studies have investigated the role of *Helicobacter pylori* infection in the development of gastric mucosa-associated lymphoid tissue (MALT) lymphoma. It is estimated that approximately 0.1% of people infected with *H. pylori* develop gastric MALT lymphoma. However, the role of the CagA antigen, the highest causative agent of *H. pylori*, in increasing the risk of gastric MALT lymphoma remains unclear and controversial. A systematic review and meta-analysis were conducted to evaluate the effect of *cagA* status on the development of gastric MALT lymphoma.

**Methods:**

All articles evaluating the status of the *cagA* gene in the development of gastric MALT lymphoma were collected using systematic searches in online databases, including PubMed, Scopus, Embase, and Google Scholar, regardless of publication date. The association between *cagA* and gastric MALT lymphoma was assessed using the odds ratio (OR) summary. In addition, a random-effects model was used in cases with significant heterogeneity.

**Results:**

A total of 10 studies met our inclusion criteria, among which 1860 patients participated. No association between *cagA* status and the development of MALT lymphoma (extranodal marginal zone B-cell lymphoma) was found in this study (OR 1.30; 0.906–1.866 with 95% CIs; *I*^2^: 45.83; *Q*-value: 12.92). Surprisingly, a meaningful association was observed between *cagA* status and diffuse large B-cell lymphoma (OR 6.43; 2.45–16.84 with 95% CIs). We also observed an inverse association between *vacA* and gastric MALT lymphoma risk (OR 0.92; 0.57–1.50 with 95% CIs).

**Conclusions:**

It seems that the infection with *cagA*-positive *H. pylori* strains does not have a meaningful effect on the gastric MALT lymphoma formation, while translocated CagA antigen into the B cells plays a crucial role in the development of diffuse large B-cell lymphoma.

## Background

*Helicobacter pylori* (*H. pylori*) is one of the most unique human pathogenic bacteria, and because of its exceptional ability to tolerate harsh stomach conditions, it colonizes the stomachs of about 4–4.5 billion people worldwide [1, 2]. Depending on environmental, socioeconomic, and health conditions, the prevalence of *H. pylori* infection varies in different geographical areas, with values of about 34% in Western countries and close to 100% in developing countries [3, 4]. Bacterial strains enter the gastric submucosa and cause chronic gastritis by evading the immune system response. However, 15–20% of people infected with *H. pylori* experience severe clinical outcomes, especially peptic ulcer disease (gastric ulcer or duodenal ulcer), chronic atrophy gastritis, and gastric adenocarcinoma, e.g., gastric cancer or mucosa-associated lymphoid tissue (MALT) lymphoma [3, 5, 6]. It is currently unknown why most people infected with *H. pylori* are asymptomatic carriers, and severe clinical outcomes are seen only in a small part of the human population. The genomic content of *H. pylori* is specific to the strain, and evaluating the role of strain virulence factors is critical [7, 8]. The cytotoxin-associated gene A (CagA) is one of the major virulence factors in this bacterium, which is encoded by the *cag* pathogenicity islands (PAIs) and is classified into four different classes based on the flanking nucleotide sequence of EPIYA motifs [9]. The *cagA* pattern in the East Asian population is usually ABD, while strains containing patterns such as ABC, ABCC, and ABCCC are isolated from Western countries [10, 11]. According to previous studies, *cagA*-positive strains are significantly present in the population with gastric ulcers and precancerous lesions [12]. Although the role of CagA protein in tumorigenesis remains unclear, according to the first hypothesis, phosphorylated CagA can phosphorylate intracellular eukaryotic proteins, particularly Src homology-2 domain-containing protein tyrosine phosphatase-2 (SHP2), and induce the hummingbird phenotype and oncogenesis by altering the normal cell signaling pathway [13]. The affinity of EPIYA-D for the binding and effect of SHP2 tyrosine phosphatase is much higher than that of EPIYA-C, so the differences are reported to be related to the fact that the prevalence of gastric cancer in East Asia is higher than that in Western countries [12, 14]. According to the second hypothesis, CagA is an immunogenic protein that stimulates the production of interleukin 8 (IL-8) and leads to the infiltration of neutrophils in the inflamed area, the production of free radicals, and DNA damage [15–18]. Accordingly, infection with this bacterium appears to increase the risk of two cancers of the digestive system, including gastric cancer and gastric MALT lymphoma [19]. According to a new classification by World Health Organization (WHO), primary gastric lymphoma (PGL) can range from extranodal marginal zone B-cell lymphoma (MALT lymphoma) to diffuse large B-cell lymphoma (DLBCL) [20]. Gastric MALT lymphoma was first identified by Isaacson and Wright in 1983 and accounts for more than 50% of gastric lymphoma, a B-cell lymphoma derived from MALT during chronic inflammation [21, 22]. In their study, Carlson et al. showed that gastritis can lead to gastric MALT lymphoma due to lymphoid hyperplasia caused by bacterium [23]. Although previous experiments had suggested that gastric MALT lymphoma is mainly associated with *H. pylori* infection, recent studies have shown a much higher rate of gastric MALT lymphoma in *H. pylori*-negative patients [24]. In a study by Asenjo et al., the global prevalence of *H. pylori* infection in DLBCL and MALT lymphoma was estimated to be about 60% and 79%, respectively [25]. Interestingly, the eradication of *H. pylori* infection is very effective in the regression of gastric MALT lymphoma; therefore, antibiotic therapy is considered the first line of treatment for gastric MALT lymphoma [26]. According to in vitro experiments, the immune response in extranodal marginal zone B-cell lymphoma is formed as a result of T cell-mediated immunity (CMI) [27, 28]. Studies have shown that the CagA antigen can be translocated to B-cell lymphocytes following the destruction of gastric mucosa during chronic gastritis [29, 30]. In B cells, this antigen prevents apoptosis through extracellular signal-regulated kinase, which in turn leads to the proliferation and immortalization of B cells and eventually MALT lymphoma [30–32]. MALT lymphoma is a complex event from primary gastric lymphoma to advanced stage called DLBCL; according to the present findings, *H. pylori* infection with host epigenetic events helps to provide favorable microenvironmental conditions for gastric lymphogenesis as a predisposing stage of precancerous lesions [33, 34]. In the gastric lumen, CagA antigen can induce pro-inflammatory responses such as neutrophil infiltration, production of reactive oxygen species (ROS), and polyclonal activation of B cells, all of which cause gastric damage and genetic instability [35, 36]; however, the role of CagA in inducing cascade of gastric precancerous lesions remains unclear. In general, despite limited information and conflicting results in some studies, events such as chronic inflammation, production of reactive oxygen species (ROS), B-cell proliferation, and genetic instability can lead to susceptibility to gastric MALT lymphoma [29, 37, 38]. In the present meta-analysis, we investigated the association between *cagA* gene status and gastric MALT lymphoma to determine the role of CagA antigen in the development of disease from MALT lymphoma to DLBCL.

## Methods

### Literature search

We conducted a comprehensive electronic search using the following online databases: PubMed, Scopus, Embase, and Google Scholar to retrieve all relevant documents printed in English up until December 2020. The search for terms was performed based on the MeSH library [39]. Accordingly, we used words such as “*Helicobacter pylori*,” “*H. pylori*,” "MALT,” “mucosa-associated lymphoid tissue,” “CagA,” “*cagA gene*”, and “cytotoxin-associated gene A”. The literature search was performed independently by two authors (MK1 and MK2) without publication date restrictions.

### Study selection

In the first stage, after initial evaluations, duplicate articles were excluded from the study, and then a reference list of each article was evaluated to avoid losing additional documents. The inclusion criteria were as follows: (1) all original, cross-sectional, case–control, and longitudinal articles related to our purpose, (2) studies on the association between *cagA* gene status and gastric MALT lymphoma; (3) studies based on standard diagnostic methods such as polymerase chain reaction (PCR), ELISA, and conventional microbiology tests; and (4) studies published in English. Exclusion criteria were as follows: (1) congress abstracts, case series, review articles, and letters to the editor; (2) articles without full text available; (3) articles published in non-English language; (4) animal studies or in vitro studies; and (5) studies with vague results and insufficient data.

### Quality assessment and data extraction

The Newcastle-Ottawa Scale (NOS) checklist was used to assess the quality of the studies (Table 1) [40–49]. The required data, including the first author, country, population sample size, number of *H. pylori* strains, diagnostic method, and frequency of *cagA*-positive strains, are listed in Table 2. All participants were divided into case (gastric MALT lymphoma) and control (gastritis or non-ulcer dyspepsia) groups.

**Table 1:** Quality assessment for eligible studies based on Newcastle-Ottawa-Scale (NOS)

Study number	First author	Year	Selection Bias assessment (Maximum 4 stars)				Comparability (Maximum 2 stars)	Outcome (Maximum 3 stars)			Total score (Maximum 10 stars)	References
			Is the case definition adequate?	Representativeness of the cases	Selection of controls	Definition of controls	Comparability of cases and controls on the basis of design or analysis	Assessment of the exposure	Same method of ascertainment for cases and controls	Non-response rate		
			Score	Score	Score	Score	Score	Score	Score	Score		
1	Jong	1996	1	1	1	1	2	1	1	1	9	[40]
2	Peng	1998	1	1	1	1	1	1	1	0	7	[41]
3	Lamarque	1999	1	0	1	0	1	1	1	0	5	[42]
4	Doorn	1999	1	0	0	1	2	1	1	1	7	[43]
5	Schmaußer	2000	1	0	1	1	1	1	1	0	6	[44]
6	Delchier	2001	1	1	1	1	2	1	1	1	9	[45]
7	Koehler	2003	1	0	1	1	1	1	1	1	7	[46]
8	Lehours	2009	1	1	1	1	2	1	1	0	8	[47]
9	Talebi	2013	1	0	1	1	1	1	1	1	7	[48]
10	Hashinaga	2016	1	1	1	1	2	1	1	0	8	[49]

**Table 2:** Characteristics of included studies

First author	Year	Country	Population size	*H*. *pylori* isolates	Diagnostic method	*cagA* positive *H*. *pylori* strains	
						Gastritis or NUD	MALT
Jong et al	1996	Netherlands	89	89	Culture-PCR	26/40	7/12
Peng et al	1998	United kingdom	123	123	PCR	17/56	37/67
Lamarque et al	1999	France	598	182	ELISA	20/54	10/17
Doorn et al	1999	Netherlands	36	36	Culture-PCR	16/24	5/12
Schmaußer et al	2000	Germany	30	30	ELISA	14/15	12/15
Delchier et al	2001	France	598	162	ELISA	22/51	29/53
Koehler et al	2003	Germany	121	91	Multiplex-PCR	27/39	15/24
Lehours et al	2009	France	79	79	PCR	22/39	21/40
Talebi et al	2013	Iran	134	128	Culture-PCR	63/74	5/28
Hashinaga et al	2016	Japan	52	52	Culture-PCR	4/22	12/12

### Statistical analysis

All statistical analyses were performed using Comprehensive Meta-Analysis software (Ver 2.2; Biostat, Englewood, NJ). The colonization rate of *cagA*-positive strains in both groups was reported as event rate (EER) with 95% confidence intervals (CIs). The impact of *cagA* gene status on the development of gastric MALT lymphoma was also measured using the odds ratio (OR) at 95% CIs. The heterogeneity between studies was assessed with the *I*^2^ > 50 test and Cochran’s *Q* Statistic *p* value > 0.05. High levels of heterogeneity were evaluated according to the random-effects model with the DerSimonian and Laird method. In contrast, the fixed-effects model, based on the Mantel–Haenszel method, was used for low levels of heterogeneity. Furthermore, publication bias was assessed using asymmetry of funnel plots, Begg’s test *p* value, and Egger’s test *p* value.

## Results

### Characteristics of selected studies

**Fig. 1:**
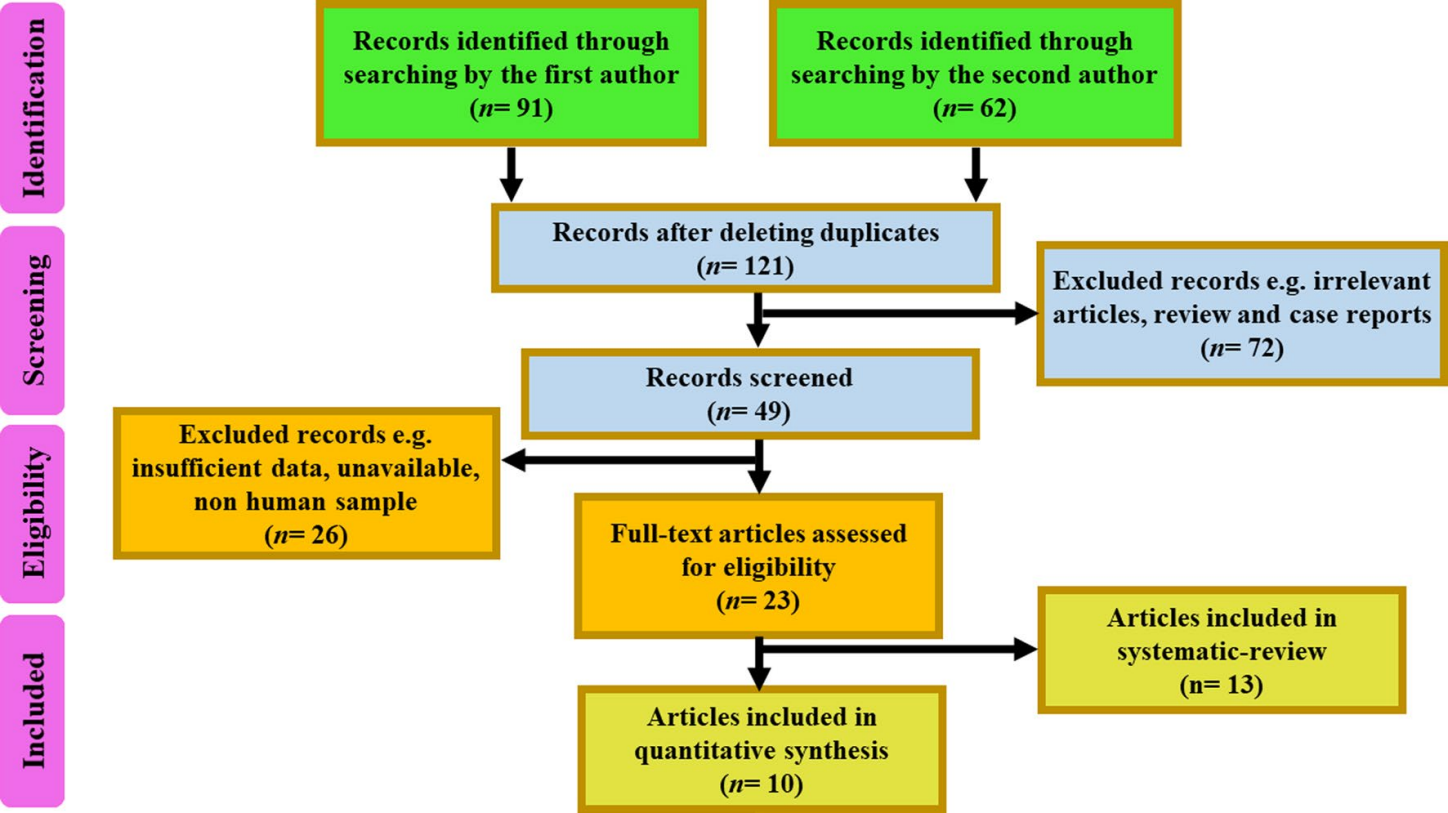
Flowchart of the article search strategy and study selection

A total of 153 articles were collected in the initial search, and finally 10 eligible articles met our criteria and were included in the current analysis. A flowchart of the article search strategy and study selection is presented in Fig. 1.

All eligible studies were conducted from 1996 to 2016, and data from 1860 patients were reviewed in these studies. Two studies were performed on the Asian population, and eight studies were conducted in Western countries. No significant relationship was observed between *cagA* status and age/sex distribution in any of the studies. In two studies, the association between *the cagA* genotype and the development of gastric MALT lymphoma was controversial [41, 48]. Unfortunately, the association between CagA EPIYA motifs and gastric MALT lymphoma had not been evaluated in all studies, so we could not investigate this association. However, in three studies, the association between *cagA* status and both extranodal marginal zone B-cell lymphoma and DLBCL forms of the MALT was investigated [41, 44, 45]. In addition, the association between *vacA* status and gastric MALT lymphoma had been assessed in six studies [41–44, 47, 49].

### Association between cagA status and susceptibility to gastric MALT lymphoma

In this study, patients were divided into cases (gastric MALT lymphoma: 280 patients) and control (gastritis/non-ulcer dyspepsia (NUD): 414 patients). The prevalence of CagA-expressing strains in patients with MALT lymphoma and gastritis/NUD patients was estimated to be 54.6% (44–64.7 with 95% CIs) and 56.4% (41.5–70.3 with 95% CIs), respectively. However, according to the subgroup analysis by different geographical regions, we found that the frequency of *cagA*-positive strains in patients with gastritis/NUD in Western countries was higher than that in Asian countries (57.6% vs. 36.5%). The results showed that the *cagA* genotype was not significantly different between Western and Asian patients with gastric MALT lymphoma.

**Fig. 2:**
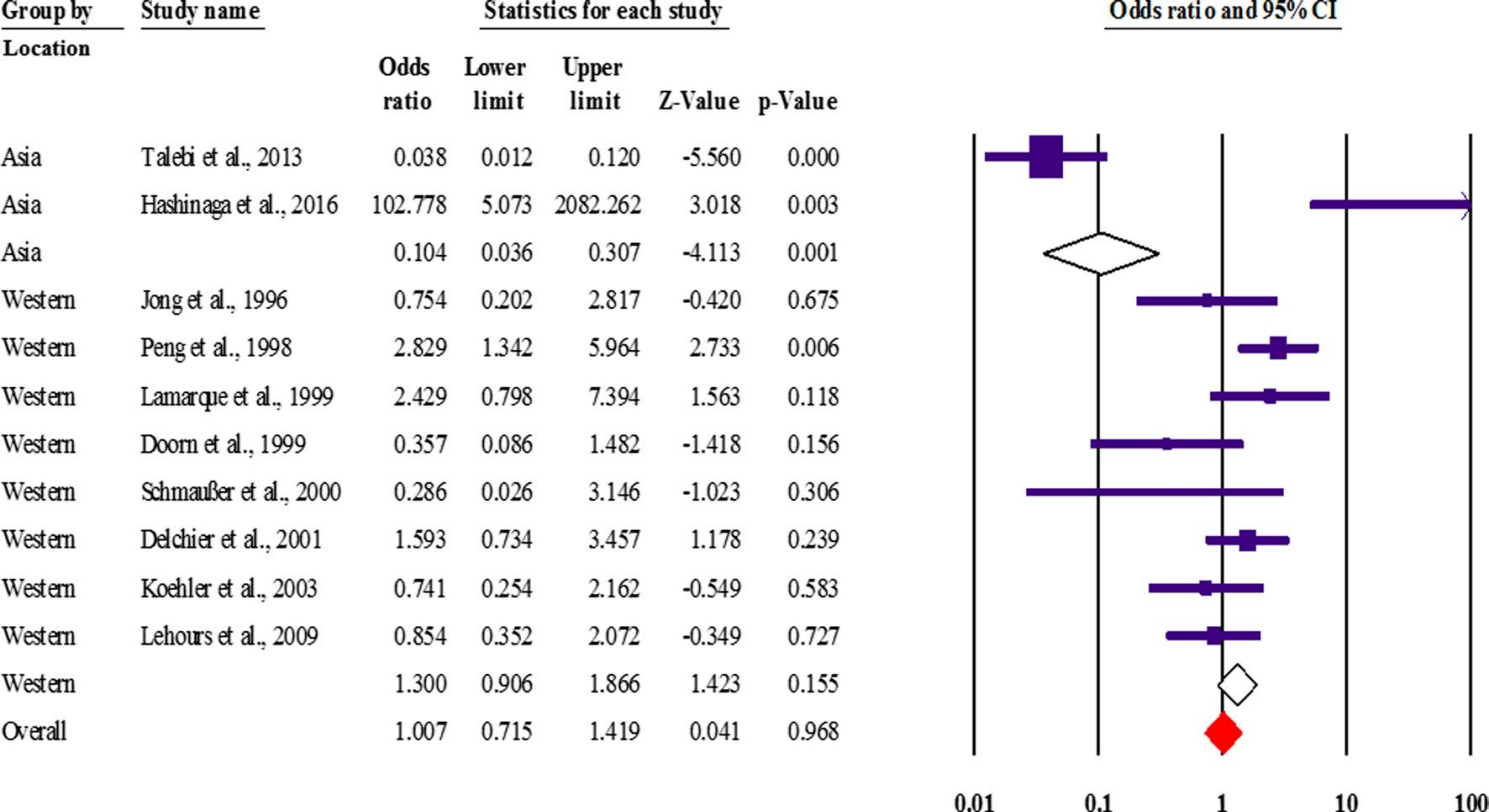
Forest plot of the meta-analysis on the potential association between cagA status and gastric MALT lymphoma with subgroup analysis based on the geographical origin of the studies

Based on the results of statistical analysis, no association was observed between *cagA* status and gastric MALT lymphoma (OR 1.00; 0.715–1.419 with 95% CIs; *p* value: 0.968; *I*^2^: 83.52; *Q*-value: 54.61; *p* value: 0.01; Egger’s *p* value: 0.36; Begg’s *p* value: 0.28) following long-term *H. pylori* infection (Fig. 2).

In the subgrouping, we found that there was an inverse association between *cagA* genotype and gastric MALT lymphoma in the Asian population (OR 0.104; 0.036–0.307 with 95% CIs; *I*^2^: 95.6; *Q*-value: 23.08; *p* value: 0.08), while there was no meaningful association between *cagA*-positive strains and the development of gastric MALT lymphoma in Western countries (OR 1.30; 0.906–1.866 with 95% CIs; *I*^2^: 45.83; *Q*-value: 12.92; *p* value: 0.58). According to the results of statistical analysis (OR 6.43; 2.45–16.84 with 95% CIs; *I*^2^: 0.00; *Q*-value: 0.6; *p* value: 0.73; Egger’s *p* value: 0.21; Begg’s *p* value: 0.21), patients infected with *cagA*-positive strains are susceptible to DLBCL (Fig. 2). We found that there was a significant inverse association between infection with VacA-expressing *H. pylori* strains and gastric MALT lymphoma (OR 0.92; 0.57–1.50 with 95% CI; *I*^2^: 32.3; *Q*-value: 7.39; *p* value: 0.1; Egger’s *p* value: 0.79; Begg’s *p* value: 0.45). We also observed a strong association between *cagA* status and the development of DLBCL, indicating the importance of this virulence factor in the immune-pathogenesis of gastric MALT lymphoma. However, further investigation is required to confirm the results of this study.

### Publication bias analysis

**Fig. 3:**
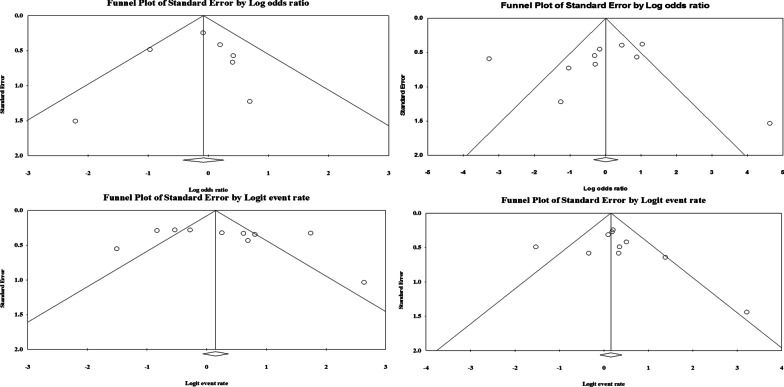
Funnel plot with 95% CIs representative the effect sizes derived from each study (logit event rate) against their corresponding standard errors

In the present study, the presence of bias in publication was evaluated using Begg’s *p* value and Egger’s *p* value. We did not observe any significant publication bias in the present study, although the funnel plot showed a slight publication bias in the eligible studies (Fig. 3).

## Discussion

Although the exact mechanism of CagA antigen at the onset of the oncogenesis process is unclear, there are two hypotheses. According to the first hypothesis, translocated CagA is phosphorylated by Src and Abl family kinases, which in turn phosphorylates intracellular eukaryotic proteins, particularly SHP2, MDM2, p53, NF-ĸB, Erk, Akt; this process induces the hummingbird phenotype and eventually oncogenesis [50, 51]. This antigen binds to several epithelial cell proteins through EPIYA motifs; intracellular SHP2 is able to bind three motifs EPIYA-B, EPIYA-C, and EPIYA-D, whereas, C-terminal Src tyrosine kinase prefers to bind EPIYA-A and EPIYA-B, as well as, Ras-GAP and Grb7 prefer to bind EPIYA-C segments [52, 53]. According to the second hypothesis, CagA is an immunogenic protein, and triggers the production of high levels of IL-8 after translocation into epithelial cells [17]. This cytokine is one of the major proinflammatory cytokines, which in turn induces the infiltration of neutrophils in infected tissues, and an excessive inflammatory response leads to the production of free radicals and DNA damage; studies have shown that the − 251 T allele in IL-8 promoter is a potential risk factor for gastric cancer [54, 55]. As mentioned, MALT lymphoma and diffuse large B-cell lymphoma are as both indolent and aggressive features of PLG, respectively. Mucosal and non-mucosal organs in human body normally lack lymphocytes, and infiltration of B-cell lymphocytes occurs as a result of chronic inflammation or autoimmune diseases (particularly Hashimoto’s thyroiditis). Gastric MALT lymphoma is the most common marginal zone lymphoma [20, 56, 57]. Today, the role of *H. pylori* infection in the development of gastric MALT lymphoma has been well-described, and recent studies have shown that eradication infection in extranodal marginal zone B-cell lymphoma can lead to lymphoma regression in 60–90% of cases [58]. The most likely hypothesis is that persistent infection with *H. pylori* can lead to inflammation and infiltration of lymphocytes into the stomach with persistent stimulation of the immune response, and active proliferation of B cells leads to the formation of lymph follicles and the onset of gastric MALT lymphoma [58]. To date, several studies have attempted to investigate the association between *cagA* status and the development of gastric MALT lymphoma, but the results are unclear [40, 41, 59]. In addition, no comprehensive meta-analysis has been performed in this area; therefore, using the available evidence, we conducted the present meta-analysis to evaluate the exact role of the CagA antigen in the development of gastric MALT lymphoma.

Overall, we did not find a meaningful relationship between *cagA* status and gastric MALT lymphoma in Western countries. Interestingly, the present analysis showed an inverse association between *cagA* status and gastric MALT lymphoma risk in the Asian population (OR 0.104; 0.036–0.307 with 95% CIs).

Previous studies indicated that early lymphomagenesis in lymphomas is a process related to CD4 + T cells stimulated by *H. pylori* antigens, and the proliferation of B-cell gastric lymphoma is dependent on CD40-mediated signaling, Th2 activities, co-stimulatory CD80, and CD86 [60–64]. Hussel et al. in their studies showed that the reduction of infiltrating T cells can significantly disrupt the effect of *H. pylori* infection on tumor B-cell proliferation [65]. Umehara et al. found that CagA could inhibit B-lymphoid cell proliferation by IL-3-dependent signaling by targeting the JAK-STAT pathway [30]. Liu et al. (2001) showed that the API2–MALT1 chimeric transcript was observed in all cases of *H. pylori*-infected gastric MALT lymphoma [66]. However, there is no correlation between *H. pylori* infection and the presence of API2–MALT1 [67]. In general, the formation of gastric MALT lymphoma is based on the dependent and independent mechanisms of *H. pylori* infection; in *H. pylori*-dependent manner (in most cases), phosphorylated CagA inhibits p53 accumulation, whereas, in *H. pylori*-independent state, genetic aberrations lead to nuclear translocation of ‏API2–MALT1 chimeric transcript and BCL10, and eventually gastric MALT lymphoma [30, 68].

**Fig. 4:**
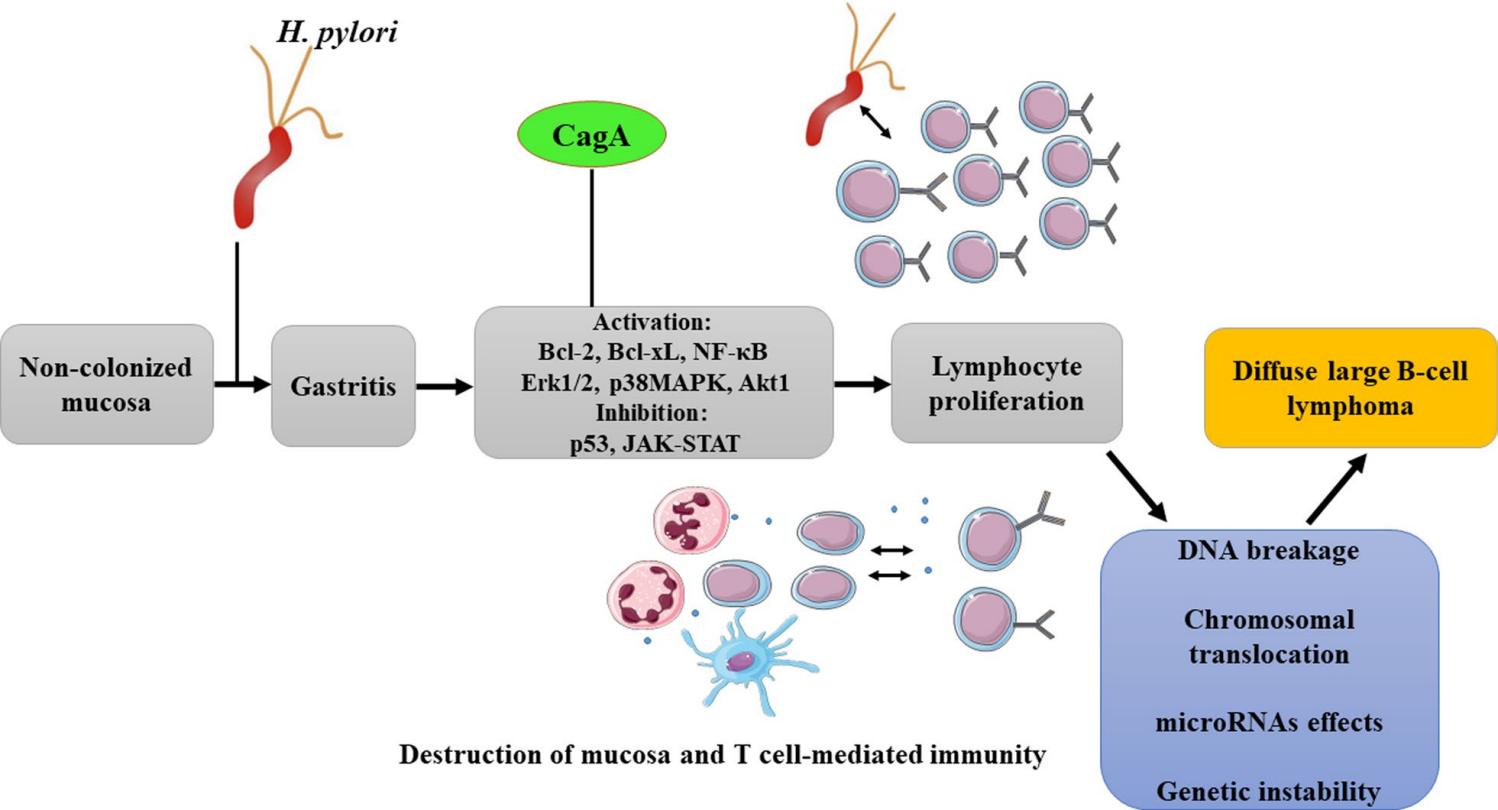
Colonization of the stomach with cagA-positive H. pylori strains and progression to DLBCL. Following long-term colonization of the bacterium in stomach mucosa, CagA protein is secreted into cells via T4SS. Upon entrance of CagA, intracellular CagA-SHP2 complex is formed. Although there is probably no association between the CagA and progression of PGL to MALT lymphoma, this complex potentially stimulates the lymphogenesis process and ultimately DLBCL by activating on ERK1, ERK2, p38MAPKs, BCL2, and NF-κB, as well as inhibiting p53 or the JAK-STAT signaling pathway. It also damages DNA and microRNA by producing reactive oxygen and nitrogen species (RONS)

Ohnishi et al. (2008) demonstrated the major role of CagA in the development of gastric and hematologic neoplasms [69]. After transfer to B-cell lymphocytes via the type 4 secretory system (T4SS), CagA, through the formation of phosphorylated CagA-SHP2 complex by affecting ERK1, ERK2, p38MAPKs, BCL2, and NF-κB, as well suppression of p53 accumulation or inhibition of the JAK-STAT signaling pathway, promotes lymphogenesis and immortalization of B-cell lymphocytes [30, 58, 70]. Evaluation of *cagA* status in patients with extranodal marginal zone B-cell lymphoma and DLBCL showed that this gene significantly increases the risk of developing DLBCL (OR 6.43; 2.45–16.84 with 95% CIs). Based on previous studies, the presence of *cagA*-positive *H. pylori* strains in patients with DLBCL is significantly higher than that in patients with extranodal marginal zone B-cell lymphoma [59]. Unfortunately, no studies have yet examined the relationship between CagA and VacA in the development of gastric MALT lymphoma, but VacA induces apoptosis by forming a vacuole and release of cytochrome c from mitochondria and appears to inhibit the development of gastric MALT lymphoma [71, 72]. The role of VacA in gastric MALT lymphoma is also controversial, and in one study, Miehlke et al. (1998) showed that the level of the *vacA* s1m1 genotype in gastric MALT lymphoma patients is high; however, Doorn et al. (1999) rejected this hypothesis [43, 73]. Although we could not assess the correlation between *cagA* and *vacA*, we observed an inverse association between the *vacA* genotype and gastric MALT lymphoma (OR 0.92; 0.57–1.50 with 95% CIs). Although *vacA* is a potent immune gene, given the fact that this protein causes apoptosis, it does not appear to play a significant role in the development of gastric MALT lymphoma [74, 75]. In general, the most likely hypothesis to describe the role of *H. pylori* in the development of gastric MALT lymphoma is that this bacterium (CagA-dependent or independent) causes chronic gastritis, resulting in the production of IL-8 and other molecules associated with neutrophil chemotaxis. Neutrophil activation leads to destruction of the gastric mucosa and close contact of CD4 + T cells with *H. pylori*, where the activity of DC and CD4 + T cells causes B cells to mature. Continuous stimulation and proliferation of B-cell lymphocytes leads to the formation of lymph follicles, in which case the patient with PGL develops to DLBCL. In other words, no *H. pylori* eradication, particularly *cagA*-positive strains, leads to the translocation of CagA into B cells. Intracellular CagA causes DNA and microRNA damage by reactive oxygen and nitrogen species (RONS), inhibition of p53, and chromosomal translocation, and ultimately the development of DLBCL (Fig. 4).

Our study had several limitations including: (1) low sample size; (2) evaluation of only English studies; (3) high heterogeneity in some cases; (4) inaccessibility to the raw data to find out EPIYA motifs CagA, as well as correlation between CagA and VacA; (5) the lack of a sensitivity analysis. The results of the study are unstable under the influence of significant heterogeneity, and more research is needed to confirm the current findings.

## Conclusion

According to the recent literature, PGL ranges from MALT lymphoma to DLBCL. In the present study, we performed a large pooled analysis to evaluate the role of *cagA* status in the pathogenesis of the gastric MALT lymphoma. Overall, based on our results, no association was found between *cagA* status and the development of MALT lymphoma. Nevertheless, CagA can stimulate lymphogenesis and leads to the contentious proliferation and immortalization of B cells; therefore, it plays an important role in the development of DLBCL of the stomach.

## Data Availability

All data generated or analyzed during this study are included in this published article and its supplementary information files.

## Abbreviations

*H. pylori*: *Helicobacter pylori*
PGL: Primary gastric lymphoma
DLBCL: Diffuse large B-cell lymphoma
MALT: Mucosa-associated lymphoid tissue
CagA: Cytotoxin-associated gene A
PCR: Polymerase chain reaction
PAIs: Pathogenicity islands
IL-8: Interleukin 8
CMI: Cell-mediated immunity
CIs: Confidence intervals
OR: Odds ratio
ROS: Reactive oxygen species
RONS: Reactive oxygen and nitrogen species
NUD: Non-ulcer dyspepsia
NOS: Newcastle-Ottawa scale

## References

[1] Kusters JG, Van Vliet AH, Kuipers EJ (2006). Pathogenesis of *Helicobacter pylori* infection. Clin Microbiol Rev.

[2] De Falco M, Lucariello A, Iaquinto S, Esposito V, Guerra G, De Luca A (2015). Molecular mechanisms of *Helicobacter pylori* pathogenesis. J Cell Physiol.

[3] Youssefi M, Tafaghodi M, Farsiani H, Ghazvini K, Keikha M (2020). Helicobacter pylori infection and autoimmune diseases; is there an association with systemic lupus erythematosus, rheumatoid arthritis, autoimmune atrophy gastritis and autoimmune pancreatitis? A systematic review and meta-analysis study. J Microbiol Immunol Infect.

[4] Hooi JK, Lai WY, Ng WK, Suen MM, Underwood FE, Tanyingoh D, Malfertheiner P, Graham DY, Wong VW, Wu JC (2017). Global prevalence of *Helicobacter pylori* infection: systematic review and meta-analysis. Gastroenterology.

[5] Karbalaei M, Keikha M (2020) Potential association between the hopQ alleles of *Helicobacter pylori* and gastrointestinal diseases: a systematic review and meta-analysis. Meta Gene 100816

[6] Šterbenc A, Jarc E, Poljak M, Homan M (2019). *Helicobacter pylori* virulence genes. World J Gastroenterol.

[7] Servetas SL, Kim A, Su H, Cha JH, Merrell DS (2018). Comparative analysis of the Hom family of outer membrane proteins in isolates from two geographically distinct regions: the United States and South Korea. Helicobacter.

[8] Gressmann H, Linz B, Ghai R, Pleissner K-P, Schlapbach R, Yamaoka Y, Kraft C, Suerbaum S, Meyer TF, Achtman M (2005). Gain and loss of multiple genes during the evolution of *Helicobacter pylori*. PLoS Genet.

[9] Keikha M, Karbalaei M (2021) EPIYA motifs of *Helicobacter pylori* cagA genotypes and gastrointestinal diseases in the Iranian population: a systematic review and meta-analysis. New Microb New Infect 100865 10.1016/j.nmni.2021.100865 PMC8066700 33912350

[10] Higashi H, Tsutsumi R, Fujita A, Yamazaki S, Asaka M, Azuma T, Hatakeyama M (2002). Biological activity of the *Helicobacter pylori* virulence factor CagA is determined by variation in the tyrosine phosphorylation sites. Proc Natl Acad Sci.

[11] Lind J, Backert S, Hoffmann R, Eichler J, Yamaoka Y, Perez-Perez GI, Torres J, Sticht H, Tegtmeyer N (2016). Systematic analysis of phosphotyrosine antibodies recognizing single phosphorylated EPIYA-motifs in CagA of East Asian-type Helicobacter pylori strains. BMC Microbiol.

[12] Li Q, Liu J, Gong Y, Yuan Y (2017) Association of CagA EPIYA-D or EPIYA-C phosphorylation sites with peptic ulcer and gastric cancer risks: a meta-analysis. Medicine 96(17) 10.1097/MD.0000000000006620 PMC5413225 28445260

[13] Yamaoka Y (2010). Mechanisms of disease: *Helicobacter pylori* virulence factors. Nat Rev Gastroenterol Hepatol.

[14] Matsunari O, Shiota S, Suzuki R, Watada M, Kinjo N, Murakami K, Fujioka T, Kinjo F, Yamaoka Y (2012). Association between *Helicobacter pylori* virulence factors and gastroduodenal diseases in Okinawa, Japan. J Clin Microbiol.

[15] Machado AMD, Figueiredo C, Touati E, Maximo V, Sousa S, Michel V, Carneiro F, Nielsen FC, Seruca R, Rasmussen LJ (2009). *Helicobacter pylori* infection induces genetic instability of nuclear and mitochondrial DNA in gastric cells. Clin Cancer Res.

[16] Karbalaei M, Khorshidi M, Sisakht-pour B, Ghazvini K, Farsiani H, Youssefi M, Keikha M (2020). What are the effects of IL-1β (rs1143634), IL-17A promoter (rs2275913) and TLR4 (rs4986790) gene polymorphism on the outcomes of infection with *H*.* pylori* within as Iranian population; a systematic review and meta-analysis. Gene Rep.

[17] Fox JG, Wang TC (2007). Inflammation, atrophy, and gastric cancer. J Clin Investig.

[18] Mégraud F, Lehours P (2007). Helicobacter pylori detection and antimicrobial susceptibility testing. Clin Microbiol Rev.

[19] Jaffe ES, Harris NL, Stein H, Isaacson PG (2008). Classification of lymphoid neoplasms: the microscope as a tool for disease discovery. Blood J Am Soc Hematol.

[20] Filip PV, Cuciureanu D, Diaconu LS, Vladareanu AM, Pop CS (2018). MALT lymphoma: epidemiology, clinical diagnosis and treatment. J Med Life.

[21] Charles ED, Green RM, Marukian S, Talal AH, Lake-Bakaar GV, Jacobson IM, Rice CM, Dustin LB (2008). Clonal expansion of immunoglobulin M+ CD27+ B cells in HCV-associated mixed cryoglobulinemia. Blood J Am Soc Hematol.

[22] Isaacson P, Wright DH (1983). Malignant lymphoma of mucosa-associated lymphoid tissue. A distinctive type of B-cell lymphoma. Cancer.

[23] Carlson SJ, Yokoo H, Vanagunas A (1996). Progression of gastritis to monoclonal B-cell lymphoma with resolution and recurrence following eradication of *Helicobacter pylori*. JAMA.

[24] Bilgilier C, Simonitsch-Klupp I, Kiesewetter B, Raderer M, Dolak W, Makristathis A, Steininger C (2016). Prevalence of clarithromycin-resistant *Helicobacter pylori* strains in gastric mucosa-associated lymphoid tissue lymphoma patients. Ann Hematol.

[25] Asenjo L, Gisbert J (2007). Prevalence of *Helicobacter pylori* infection in gastric MALT lymphoma: a sistematic review. Rev Esp Enferm Dig.

[26] Gisbert J, Calvet X (2011). common misconceptions in the management of *Helicobacter pylori*-associated gastric MALT-lymphoma. Aliment Pharmacol Ther.

[27] Lim KH, Yang Y, Staudt LM (2012). Pathogenetic importance and therapeutic implications of NF-κB in lymphoid malignancies. Immunol Rev.

[28] Fischbach W, Goebeler-Kolve M, Dragosics B, Greiner A, Stolte M (2004). Long term outcome of patients with gastric marginal zone B cell lymphoma of mucosa associated lymphoid tissue (MALT) following exclusive *Helicobacter pylori* eradication therapy: experience from a large prospective series. Gut.

[29] Lin W-C, Tsai H-F, Kuo S-H, Wu M-S, Lin C-W, Hsu P-I, Cheng A-L, Hsu P-N (2010). Translocation of *Helicobacter pylori* CagA into Human B lymphocytes, the origin of mucosa-associated lymphoid tissue lymphoma. Can Res.

[30] Umehara S, Higashi H, Ohnishi N, Asaka M, Hatakeyama M (2003). Effects of *Helicobacter pylori* CagA protein on the growth and survival of B lymphocytes, the origin of MALT lymphoma. Oncogene.

[31] Chen CY, Wang FY, Wan HJ, Jin XX, Wei J, Wang ZK, Liu C, Lu H, Shi H, Li DH (2013). Amino acid polymorphisms flanking the EPIYA-A motif of H elicobacter pylori CagA C-terminal region is associated with gastric cancer in East China: experience from a single center. J Dig Dis.

[32] Krisch LM, Posselt G, Hammerl P, Wessler S (2016). CagA phosphorylation in *Helicobacter pylori*-infected B cells is mediated by the nonreceptor tyrosine kinases of the Src and Abl families. Infect Immun.

[33] Valenzuela MA, Canales J, Corvalán AH, Quest AF (2015). *Helicobacter pylori*-induced inflammation and epigenetic changes during gastric carcinogenesis. World J Gastroenterol.

[34] Craig VJ, Cogliatti SB, Rehrauer H, Wündisch T, Müller A (2011). Epigenetic silencing of microRNA-203 dysregulates ABL1 expression and drives Helicobacter-associated gastric lymphomagenesis. Can Res.

[35] Bagheri V, Memar B, Momtazi AA, Sahebkar A, Gholamin M, Abbaszadegan MR (2018). Cytokine networks and their association with *Helicobacter pylori* infection in gastric carcinoma. J Cell Physiol.

[36] Touati E, Michel V, Thiberge J-M, Wuscher N, Huerre M, Labigne A (2003). Chronic *Helicobacter pylori* infections induce gastric mutations in mice. Gastroenterology.

[37] Peng JC, Zhong L, Ran ZH (2015). Primary lymphomas in the gastrointestinal tract. J Dig Dis.

[38] Wang H-P, Zhu Y-L, Shao W (2013). Role of *Helicobacter pylori* virulence factor cytotoxin-associated gene A in gastric mucosa-associated lymphoid tissue lymphoma. World J Gastroenterol.

[39] Askari P, Karbalaei M, Ghazvini K, Keikha M (2021) Severe clinical outcomes of infection with babA2-positive Helicobacter pylori strains in the Iranian population: a systematic review and meta-analysis. Meta Gene 100911

[40] De Jong D, Van Der Hulst RW, Pals G, Van Dijk WC, Van Der Ende A, Tytgat GN, Taal BG, Boot H (1996). Gastric non-Hodgkin lymphomas of mucosa-associated lymphoid tissue are not associated with more aggressive *Helicobacter pylori* strains as identified by CagA. Am J Clin Pathol.

[41] Peng H, Ranaldi R, Diss TC, Isaacson PG, Bearzi I, Pan L (1998). High frequency of CagA+ *Helicobacter pylori* infection in high-grade gastric MALT B-cell lymphomas. J Pathol.

[42] Lamarque D, Gilbert T, Roudot-Thoraval F, Deforges L, Chaumette MT, Delchier JC (1999). Seroprevalence of eight *Helicobacter pylori* antigens among 182 patients with peptic ulcer, MALT gastric lymphoma or non-ulcer dyspepsia. Higher rate of seroreactivity against CagA and 35-kDa antigens in patients with peptic ulcer originating from Europe and Africa. Eur J Gastroenterol Hepatol.

[43] van Doorn NE, Namavar F, van Doorn L-J, Durrani Z, Kuipers EJ, Vandenbroucke-Grauls CM (1999). Analysis of vacA, cagA, and IS 605 genotypes and those determined by PCR amplification of DNA between repetitive sequences of *Helicobacter pylori* strains isolated from patients with nonulcer dyspepsia or mucosa-associated lymphoid tissue lymphoma. J Clin Microbiol.

[44] Schmaußer B, Eck M, Greiner A, Kraus M, Müller-Hermelink HK (2000). Mucosal humoral immune response to CagA shows a high prevalence in patients with gastric MALT-type lymphoma. Virchows Arch.

[45] Delchier J-C, Lamarque D, Levy M, Tkoub EM, Copie-Bergman C, Deforges L, Chaumette M-T, Haioun C (2001). Helicobacter pylori and gastric lymphoma: high seroprevalence of CagA in diffuse large B-cell lymphoma but not in low-grade lymphoma of mucosa-associated lymphoid tissue type. Am J Gastroenterol.

[46] Koehler C, Mues M, Dienes H, Kriegsmann J, Schirmacher P, Odenthal M (2003). Helicobacter pylori genotyping in gastric adenocarcinoma and MALT lymphoma by multiplex PCR analyses of paraffin wax embedded tissues. Mol Pathol.

[47] Lehours P, Zheng Z, Skoglund A, Mégraud F, Engstrand L (2009). Is there a link between the lipopolysaccharide of Helicobacter pylori gastric MALT lymphoma associated strains and lymphoma pathogenesis?. PLoS ONE.

[48] Abadi ATB, Ghasemzadeh A, Mobarez AM (2013). Low frequency of cagA-positive *Helicobacter pylori* strains isolated from Iranian patients with MALT lymphoma. Intern Emerg Med.

[49] Hashinaga M, Suzuki R, Akada J, Matsumoto T, Kido Y, Okimoto T, Kodama M, Murakami K, Yamaoka Y (2016). Differences in amino acid frequency in CagA and VacA sequences of *Helicobacter pylori* distinguish gastric cancer from gastric MALT lymphoma. Gut Pathogens.

[50] Yong X, Tang B, Li B-S, Xie R, Hu C-J, Luo G, Qin Y, Dong H, Yang S-M (2015). Helicobacter pylori virulence factor CagA promotes tumorigenesis of gastric cancer via multiple signaling pathways. Cell Commun Signal.

[51] Sougleri IS, Papadakos KS, Zadik MP, Mavri-Vavagianni M, Mentis AF, Sgouras DN (2016). *Helicobacter pylori* CagA protein induces factors involved in the epithelial to mesenchymal transition (EMT) in infected gastric epithelial cells in an EPIYA-phosphorylation-dependent manner.

[52] Hatakeyama M (2004). Oncogenic mechanisms of the Helicobacter pylori CagA protein. Nat Rev Cancer.

[53] Selbach M, Paul FE, Brandt S, Guye P, Daumke O, Backert S, Dehio C, Mann M (2009). Host cell interactome of tyrosine-phosphorylated bacterial proteins. Cell Host Microbe.

[54] Lee W-P, Tai DI, Lan KH, Li AFY, Hsu HC, Lin EJ, Lin YP, Sheu ML, Li CP, Chang FY (2005). The -251T allele of the interleukin-8 promoter is associated with increased risk of gastric carcinoma featuring diffuse-type histopathology in Chinese population. Clin Cancer Res.

[55] Taguchi A, Ohmiya N, Shirai K, Mabuchi N, Itoh A, Hirooka Y, Niwa Y, Goto H (2005). Interleukin-8 promoter polymorphism increases the risk of atrophic gastritis and gastric cancer in Japan. Cancer Epidemiol Prevent Biomark.

[56] Ye H, Liu H, Attygalle A, Wotherspoon AC, Nicholson AG, Charlotte FDR, Leblond V, Speight P, Goodlad J, Lavergne-Slove A (2003). Variable frequencies of t (11; 18)(q21; q21) in MALT lymphomas of different sites: significant association with CagA strains of H pylori in gastric MALT lymphoma. Blood.

[57] Keikha M, Karbalaei M (2021). Correlation between the geographical origin of *Helicobacter pylori* homB-positive strains and their clinical outcomes: a systematic review and meta-analysis. BMC Gastroenterol.

[58] Floch P, Mégraud F, Lehours P (2017). *Helicobacter pylori* strains and gastric MALT lymphoma. Toxins.

[59] Kuo S, Yeh K, Chen L, Lin C, Hsu P, Hsu C, Wu M, Tzeng Y, Tsai H, Wang H (2014). Helicobacter pylori-related diffuse large B-cell lymphoma of the stomach: a distinct entity with lower aggressiveness and higher chemosensitivity. Blood Cancer J.

[60] Farinha P, Gascoyne RD (2005). *Helicobacter pylori* and MALT lymphoma. Gastroenterology.

[61] Mueller A, O'Rourke J, Chu P, Chu A, Dixon MF, Bouley DM, Lee A, Falkow S (2005). The role of antigenic drive and tumor-infiltrating accessory cells in the pathogenesis of helicobacter-induced mucosa-associated lymphoid tissue lymphoma. Am J Pathol.

[62] Mimuro H, Suzuki T, Nagai S, Rieder G, Suzuki M, Nagai T, Fujita Y, Nagamatsu K, Ishijima N, Koyasu S (2007). *Helicobacter pylori* dampens gut epithelial self-renewal by inhibiting apoptosis, a bacterial strategy to enhance colonization of the stomach. Cell Host Microbe.

[63] Carreras J, Lopez-Guillermo A, Fox BC, Colomo L, Martinez A, Roncador G, Montserrat E, Campo E, Banham AH (2006). High numbers of tumor-infiltrating FOXP3-positive regulatory T cells are associated with improved overall survival in follicular lymphoma. Blood.

[64] D’Elios MM, Amedei A, Del Prete G (2003). Helicobacter pylori antigen-specific T-cell responses at gastric level in chronic gastritis, peptic ulcer, gastric cancer and low-grade mucosa-associated lymphoid tissue (MALT) lymphoma. Microbes Infect.

[65] Hussell T, Isaacson PG, Spencer J, Crabtree J (1993). The response of cells from low-grade B-cell gastric lymphomas of mucosa-associated lymphoid tissue to *Helicobacter pylori*. Lancet.

[66] Liu H, Ruskon-Fourmestraux A, Lavergne-Slove A, Ye H, Molina T, Bouhnik Y, Hamoudi RA, Diss TC, Dogan A, Megraud F (2001). Resistance of t (11; 18) positive gastric mucosa-associated lymphoid tissue lymphoma to *Helicobacter pylori* eradication therapy. The Lancet.

[67] Baens M, Maes B, Steyls A, Geboes K, Marynen P, De Wolf-Peeters C (2000). The product of the t (11; 18), an API2-MLT fusion, marks nearly half of gastric MALT type lymphomas without large cell proliferation. Am J Pathol.

[68] Kuo S, Chen L, Lin C, Wu M, Hsu P, Tsai H, Chu C, Tzeng Y, Wang H, Yeh K (2013). Detection of the *Helicobacter pylori* CagA protein in gastric mucosa-associated lymphoid tissue lymphoma cells: clinical and biological significance. Blood Cancer J.

[69] Ohnishi N, Yuasa H, Tanaka S, Sawa H, Miura M, Matsui A, Higashi H, Musashi M, Iwabuchi K, Suzuki M (2008). Transgenic expression of *Helicobacter pylori* CagA induces gastrointestinal and hematopoietic neoplasms in mouse. Proc Natl Acad Sci.

[70] Zhu Y, Wang C, Huang J, Ge Z, Dong Q, Zhong X, Su Y, Zheng S (2007). The *Helicobacter pylori* virulence factor CagA promotes Erk1/2-mediated Bad phosphorylation in lymphocytes: a mechanism of CagA-inhibited lymphocyte apoptosis. Cell Microbiol.

[71] Matsumoto A, Isomoto H, Nakayama M, Hisatsune J, Nishi Y, Nakashima Y, Matsushima K, Kurazono H, Nakao K, Hirayama T (2011). *Helicobacter pylori* VacA reduces the cellular expression of STAT3 and pro-survival Bcl-2 family proteins, Bcl-2 and Bcl-X L, leading to apoptosis in gastric epithelial cells. Dig Dis Sci.

[72] Palframan SL, Kwok T, Gabriel K (2012). Vacuolating cytotoxin A (VacA), a key toxin for *Helicobacter pylori* pathogenesis. Front Cell Infect Microbiol.

[73] Miehlke S, Meining A, Morgner A, Bayerdörffer E, Lehn N, Stolte M, Graham DY, Go MF (1998). Frequency of vacA genotypes and cytotoxin activity in Helicobacter pylori associated with low-grade gastric mucosa-associated lymphoid tissue lymphoma. J Clin Microbiol.

[74] Ihan A, Pinchuk IV, Beswick EJ (2012). Inflammation, immunity, and vaccines for *H elicobacter* pylori Infection. Helicobacter.

[75] Cover TL, Krishna US, Israel DA, Peek RM (2003). Induction of gastric epithelial cell apoptosis by *Helicobacter pylori* vacuolating cytotoxin. Can Res.

